# Asthma Diagnosis: The Changing Face of Guidelines

**DOI:** 10.1007/s41030-019-0093-y

**Published:** 2019-07-01

**Authors:** Sarah M. Drake, Angela Simpson, Stephen J. Fowler

**Affiliations:** 1 Division of Infection, Immunity and Respiratory Medicine, School of Biological Sciences, University of Manchester, Manchester, UK; 2 Manchester Academic Health Science Centre and NIHR Manchester Biomedical Research Centre, Manchester University Hospitals NHS Foundation Trust, Manchester, UK

**Keywords:** Asthma, Diagnosis, Endotype, Guidelines, Impulse oscillometry (IOS), Multiple-breath washout (MBW), Paediatric, Phenotype, Treatable trait, Volatile organic compounds (VOCs)

## Abstract

Asthma, the most common chronic respiratory disease, is frequently misdiagnosed, and accounts for a significant proportion of healthcare expenditure. This has driven the National Institute for Health and Care Excellence (NICE) in the United Kingdom (UK) to produce recent guidance; in places, this contrasts to that of the British Thoracic Society/Scottish Intercollegiate Guideline Network (BTS/SIGN), which have been producing their own guidance since 2003. Here we review the history of asthma diagnostic guidelines, and compare and review the evidence behind them, in adults and in children. We discuss the definitions of asthma and how these drive the concepts behind diagnostic strategies. We anticipate future directions in asthma diagnosis which will take into account the concepts of personalised medicine and disease endotypes. We also consider the utility of tests in use now and in the future, in particular novel tests relating to small airway inflammation and obstruction.

## History of Asthma and Diagnostic Guidelines

Asthma is the most common chronic respiratory disease affecting people from childhood through to adulthood [21]. It is a characterised by variable expiratory airflow limitation, classically presenting with episodes of wheeze, shortness of breath, chest tightness and/or cough [40]. Asthma presents a significant global health burden. The World Health Organization (WHO) published estimates suggesting that more than 235 million people worldwide are affected by asthma, and that over 380,000 deaths were attributed to asthma over a 12-month period [37]. In the United Kingdom (UK), on average three people will die from asthma every day [44]. Asthma has been shown to be underdiagnosed across all countries irrespective of the level of development [37]. In addition, a large population study in Canada demonstrated that up to 33% of people may have been incorrectly diagnosed and treated for asthma; this group were more likely to have received their initial diagnosis in the absence of objective testing [1]. As both over- and under-diagnosis are significant concerns, accurate diagnosis is vital in order to optimise health and improve quality of life and survival.

In order to establish a diagnosis we must first understand asthma. The term originates from the Greek verb “aazein”, meaning to pant or exhale with an open mouth [32]. Historically the word “asthma” was first documented as a medical term in the *Corpus Hippocraticum* (460–370 bc). The term was used to indicate a form of difficult breathing; it was a descriptive word to denote a symptom that was more severe than dyspnoea but less severe than orthopnoea [27]. Over time the word evolved to become the name of a disease that is now embedded within modern medical textbooks. Despite asthma being both well acknowledged and widespread, there was no guidance available on how to best diagnose or treat the disease until an epidemic of asthma deaths emerged in the 1960s [41]. The costs of not recognising and treating asthma correctly triggered a progressive increase in asthma-related research, driven by public health and health economics.

**Fig. 1:**
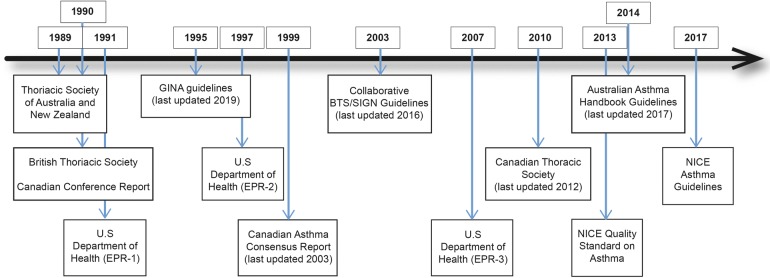
Evolution of asthma guidelines

The first published national asthma guidelines were developed by the Thoracic Society of Australia and New Zealand in 1989 [45] (Fig. 1). These were closely followed by guidance from the British Thoracic Society [22] and a Canadian practical guideline report [24] in 1990. The US Department of Health guidelines (EPR-1) followed in 1991 [23], at the same time the International Study of Asthma and Allergies in Childhood (ISAAC) program was commenced, with a view to study the aetiology of asthma [5]. Over subsequent years, more comprehensive national and international guidance has evolved, and in parallel there has been a decline in the age-adjusted death rate attributed to asthma. Despite this, asthma-related mortality overall remains high. This has been attributed to an aging population [19]; however, it would be naïve to assume that this is the only explanation, with ongoing debate still concerning optimal diagnostic and management strategies for this common disease.

### Compliance with Ethics Guidelines

This article is based on previously conducted studies and does not contain any studies with human participants or animals performed by any of the authors.

## The Changing Face of Asthma Diagnosis

Recent literature has taken us back to thinking about asthma by its original descriptive and symptom-focused roots rather than describing a discrete disease entity [13, 38]. There is a drive to determine the underlying cause of the “symptom” asthma in an individual, acknowledging that there are likely multiple aetiologies which may require different diagnostic and management pathways. A popular analogy compares asthma with “anaemia” [38], both terms being used to describe manifestations of diseases reflecting several pathophysiological mechanisms. Whilst the analogy is useful in reflecting the potential complexity of asthma in an individual, its shortcomings exemplify one of the major issues in asthma care: whilst anaemia can be diagnosed with a simple blood test (i.e. haemoglobin level), no such single objective test exists to diagnose asthma.

Several approaches have emerged towards deconstructing asthma and categorising patients either by the underlying disease process or by specific clinical characteristics. Endotypes refer to distinct groups with well-defined cellular or molecular biomarkers and a discrete underlying pathophysiology [16]. Evolution of endotypes has in part been a “reactive” process secondary to advances in asthma treatments, which are being developed to act upon specific pathophysiological abnormalities. There is now a need to highlight the underlying cause of the asthma symptoms experienced by a patient in order to prescribe the most effective drug. It would be neither appropriate nor cost-effective to treat all patients that have the “symptom” asthma with a targeted drug, unless it acts specifically upon that patient's underlying pathophysiological abnormality. The process of deconstructing asthma into the underlying diseases by endotyping is important, but it is likely to evolve slowly over time as our understanding of airway pathophysiology continues to advance. In the interim, defining a universal diagnostic pathway will be challenging; it is likely that multiple pathways with linked biomarkers may be required in the future.

Another way of deconstructing asthma is through phenotypes, defined by observable symptoms or disease characteristics. Phenotyping is possible through assessment of clinical, functional, radiological or biological parameters [3]. This is distinct from endotypes, which requires knowledge of the underlying cellular or molecular pathology. Hence, identifying the phenotype may help to select drugs that improve the observed clinical presentation, whereas endotype-driven therapy will target an underlying mechanism directly.

A linked concept is that of treatable traits, defined as observable components that can be modified to improve well-being [3, 33, 43]. The concept can encompass both classification systems and is perhaps a more clinically useful way to classify asthma. It can be illustrated by the aforementioned comparison with “anaemia”. A patient who presents with breathlessness due to anaemia may benefit symptomatically from a blood transfusion, irrespective of the underlying disease. Likewise, a patient who presents with breathlessness and wheeze due to bronchoconstriction will likely benefit from a bronchodilator inhaler irrespective of the underlying mechanism.

With the emergence of phenotypes and endotypes and observation of their overlap, attempts have been made to unravel these in order to provide a more accurate prediction of an individual's prognosis and determine the most effective treatment plan [2]. Whilst continuing to explore the underlying endotypes and origins of asthma, an interim model is required for the present day. The “treatable trait” model is both easier to understand and currently more clinically useful. Common treatable traits can be found in Table 1. Identifying some of these traits within the diagnostic algorithms has the potential to enable early and appropriate therapeutic management of asthma. *The Lancet* asthma commission [38] also advocated deconstructing asthma characteristics into treatable traits, supporting the concept of a precision approach and opposing the current “one-size-fits-all” approach to asthma management.

**Table 1:** Examples of “treatable traits” that could prompt targeted intervention in asthma

Pulmonary	Symptom-based	Wheeze Cough (productive/non-productive) Breathlessness
	Modifiable exposures	Allergens Bacterial infection Viral infection Exercise Occupational
	Functional	Variable airflow limitation Bronchial hyperresponsiveness Fixed airflow obstruction
	Radiological	Air trapping Airway wall thickening
	Biological	Elevated FeNO Blood/airway eosinophilia Elevated total/specific IgE
	Pathological	Airway remodelling
Extra pulmonary		Obesity Obstructive sleep apnoea Rhinosinusitis Eczema Gastro-oesophageal reflux disease Dysfunctional breathing pattern Inducible laryngeal obstruction
Behavioural/psychosocial		Anxiety Depression Smoking Poor medication adherence

The result of this evolving perception of asthma, and also the recognition that asthma is inadequately diagnosed across the world, has triggered recent changes in diagnostic guidelines. Guidelines have started to encompass more objective tests within the diagnostic algorithms. These objective tests will assist in grouping patients with the “symptom” asthma and enabling earlier exposure to appropriate treatments. However, different national and international diagnostic algorithms currently present conflicting advice.

### The Changing Face of Asthma Diagnostic Guidelines: The United Kingdom

At present, two national guidelines are available for treating asthma in the UK, both aiming to recommend the best approach for diagnosing (and treating) asthma, but contradicting one another in several key areas. These guidelines, produced by the British Thoracic Society in partnership with the Scottish Intercollegiate Guidelines Network (BTS/SIGN) [36], which cover the whole of the UK, and by the National Institute for Health and Care Excellence (NICE) [17], which cover only England, have led to confusion and significant concerns amongst healthcare professionals [20, 26, 42].

**Fig. 2:**
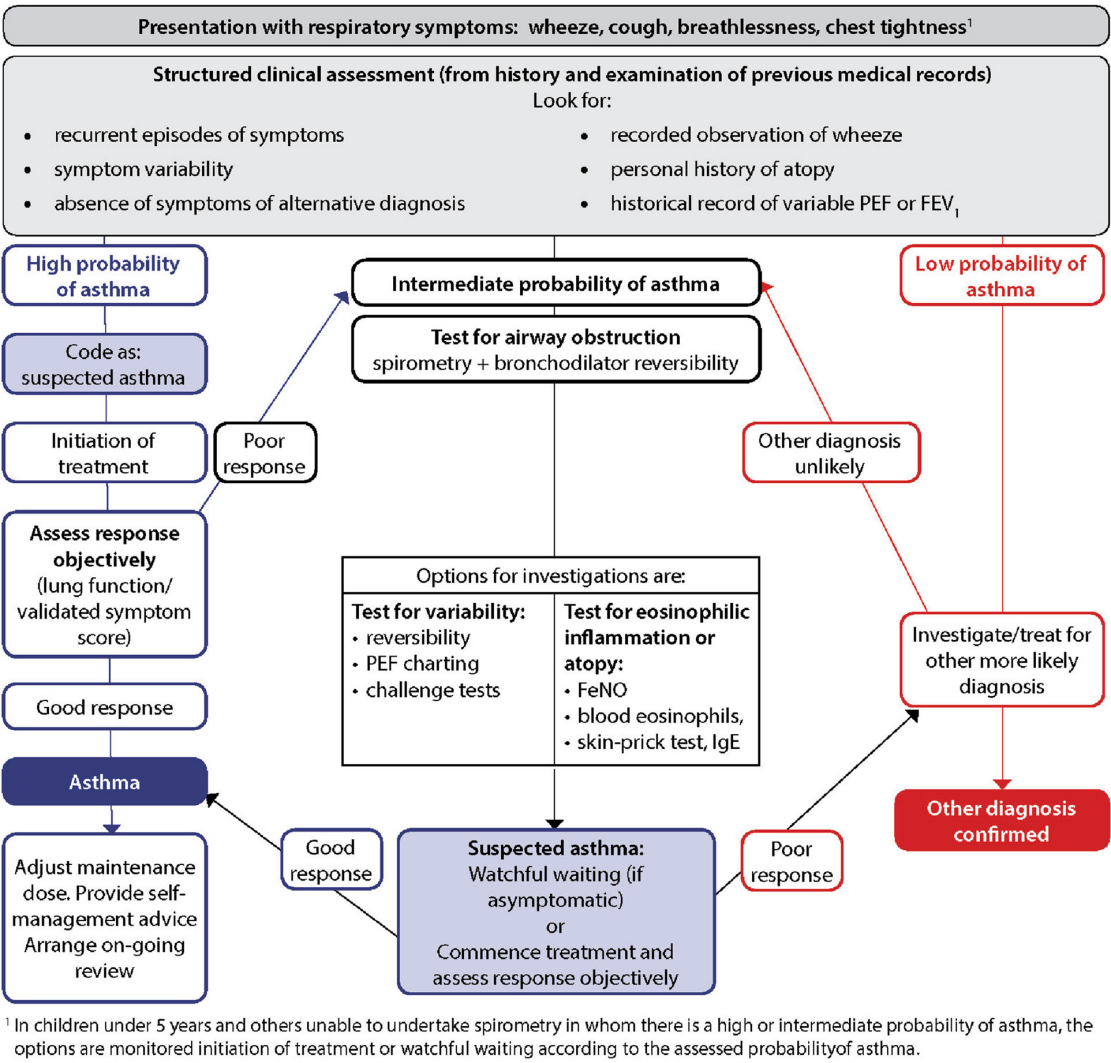
BTS diagnostic algorithm [36] (This figure is reproduced from the BTS/SIGN British Guideline on the Management of Asthma by kind permission of the British Thoracic Society)

Until recently, the asthma guideline produced by BTS/SIGN (see Fig. 2) [36] has been widely accepted in the UK [26]. The first formal BTS guidelines were published in 1990. The guidelines evolved over the subsequent decade, and in 2003 the introduction of a more evidence-based methodology was formally introduced when BTS joined with SIGN to produce the British Guideline on the Management of Asthma. This guideline was formed in collaboration with Asthma UK, the Royal College of Physicians of London and the Royal College of Paediatrics and Child Health amongst others [36]. The latest version, updated in 2016, provides recommendations for asthma diagnosis in children and adults. The guideline recommends a clinical diagnosis based predominantly upon physician assessment and encourages the use of objective investigation to demonstrate variable airflow obstruction or bronchial hyperresponsiveness (BHR). However, objective tests are not a requirement for diagnosis. The guideline recommends that a patient having a “high probability” of asthma based upon structured clinical assessment alone is sufficient to commence asthma treatment and subsequently to confirm the diagnosis if there is a perceived treatment response.

A “high probability” of asthma is supported by evidence of episodic symptoms, auscultated wheeze, history of atopy and no suggestion of an alternative diagnosis. In this case, objective testing is not required, even though it has previously been demonstrated that diagnosing asthma in the absence of objective tests was associated with over-diagnosis of asthma [1]. Furthermore, by following this algorithm, the diagnosis (through both the “intermediate probability” and “high probability” routes) is based on response to a trial of low- to medium-dose inhaled corticosteroid treatment, a premise that could lead to diagnostic error. First, asthma and “corticosteroid-responsive respiratory symptoms” are overlapping but different entities. Second, a positive or negative response to treatment, whether based on symptoms only or including lung function, is not a robust test. Major causes of a positive response other than corticosteroid-responsive disease include placebo response (usually very high in studies of inhaled pharmacotherapy) and natural variability in the symptoms; the patients may well have presented at a nadir (for example following a recent exacerbation triggered by a viral infection or allergen exposure), which then could have improved spontaneously at the time of consultation. Conversely, a negative response could be due to poor adherence to regular therapy or to progression of disease.

**Fig. 3:**
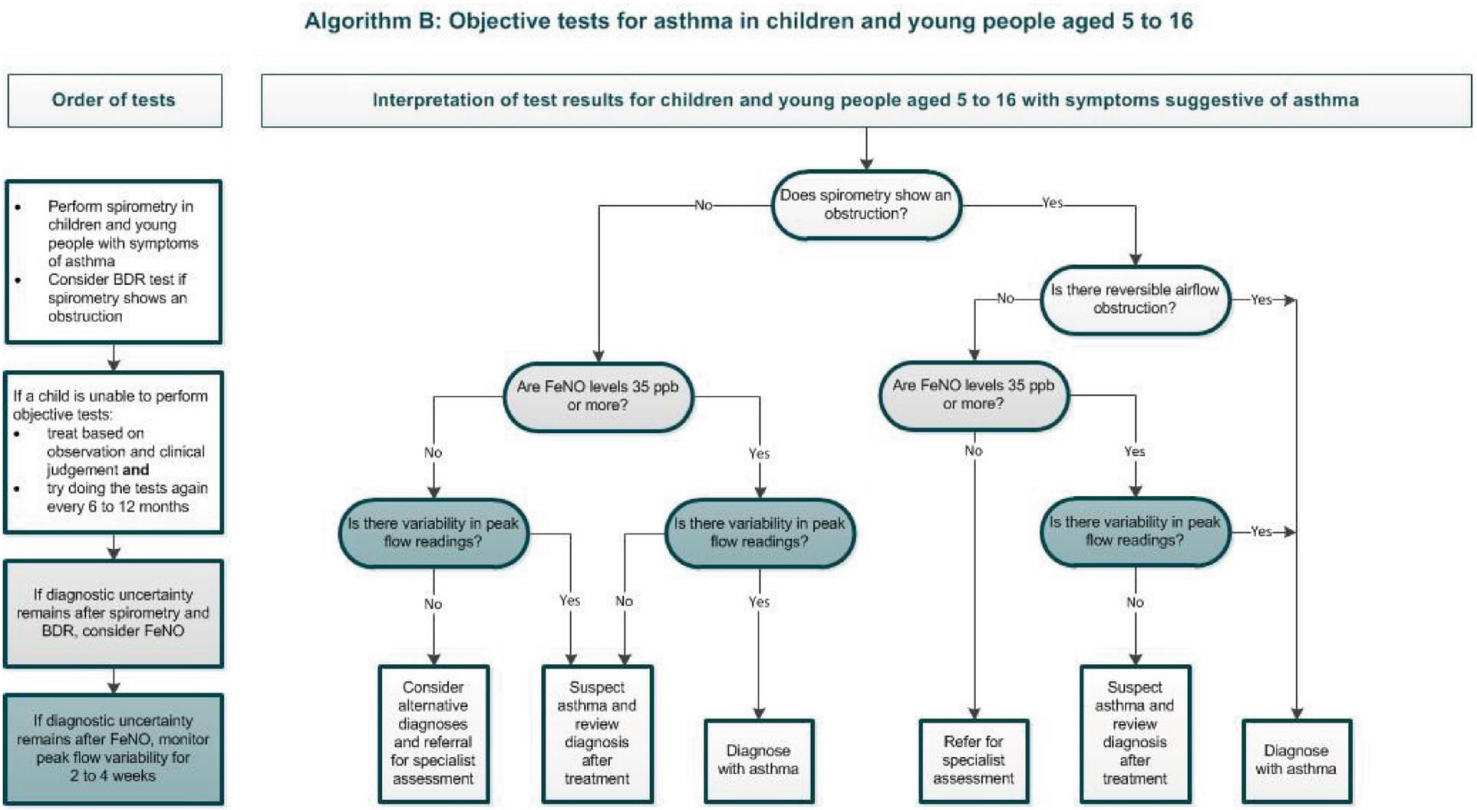
NICE diagnostic algorithm in children [17]: https://www.nice.org.uk/guidance/ng80/resources/algorithm-b-objective-tests-for-asthma-in-children-and-young-people-aged-5-to-16-pdf-4656176750. Asthma: diagnosis, monitoring and chronic asthma management (NG80)

**Fig. 4:**
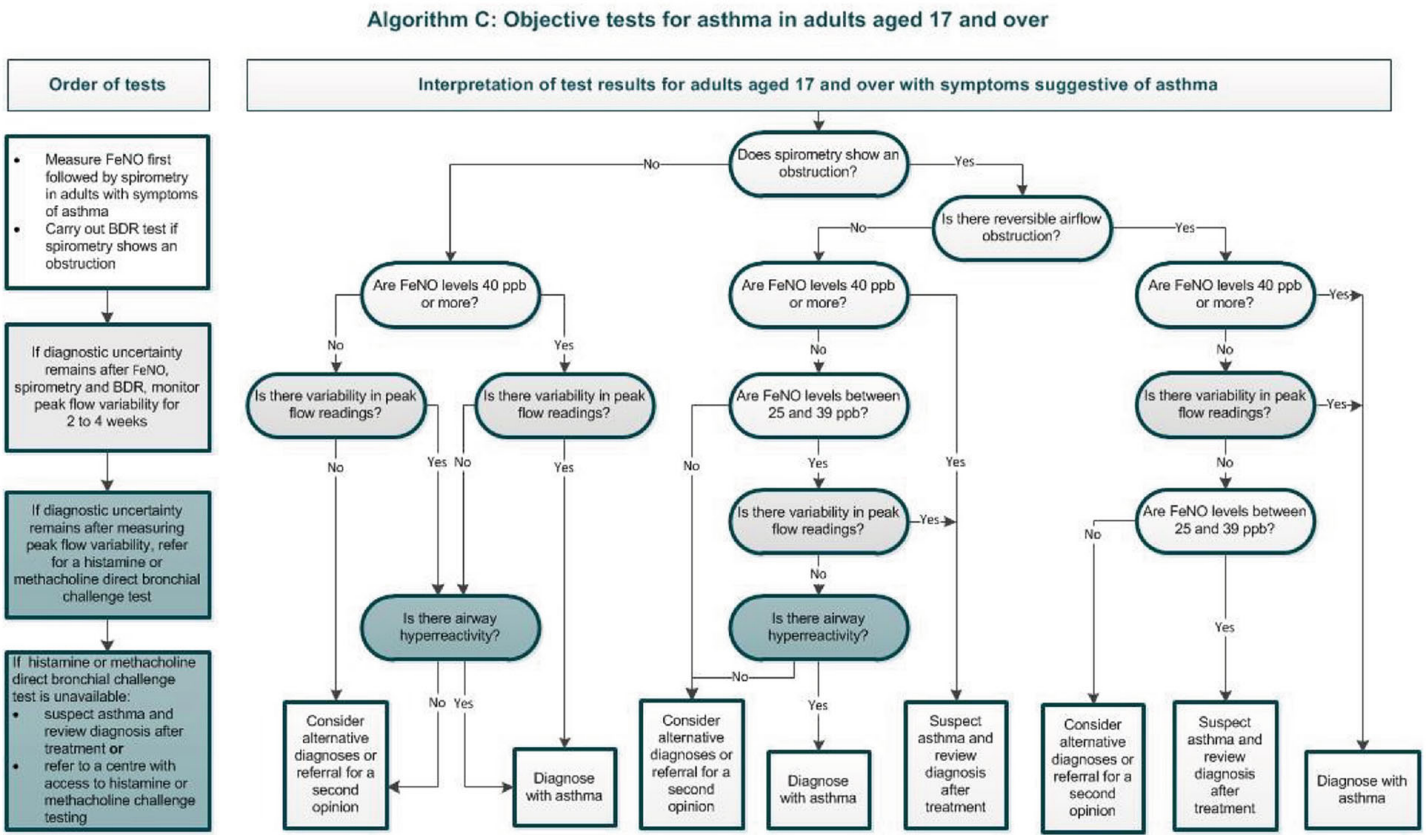
NICE diagnostic algorithm in adults [17]: https://www.nice.org.uk/guidance/ng80/resources/algorithm-c-objective-tests-for-asthma-in-adults-aged-17-and-over-pdf-4656176751

Another recent guideline on diagnosis and management of asthma was produced by NICE (see Figs. 3, 4) [17]. NICE methodology differs from BTS/SIGN in that, in addition to an evidence-based approach, the guideline places an emphasis on a health economics analysis. NICE guidelines critique the evidence on asthma diagnosis using clinical assessment alone (a strategy employed in one pathway of the BTS/SIGN algorithm), concluding that this approach was found to have poor specificity, and is likely contributing to over-diagnosis [17]. The guideline therefore recommends compulsory objective investigations for asthma diagnosis. Perhaps due to an emphasis on health economy, NICE recommend using an algorithm with sequential tests. The algorithm includes tests of airflow obstruction (i.e. spirometry), bronchodilator reversibility (BDR), airway inflammation (i.e. fractional exhaled nitric oxide (FeNO)) and airflow variability, plus bronchial challenge tests if results are inconclusive. The lack of a single gold-standard test necessitates combination testing, and developing a reliable diagnostic pathway with as few investigations as possible makes sense, although the diagnostic performance of these tests in the sequence recommended has not been validated. The health economics weighting could perhaps mean that tests such as peak expiratory flow variability (PEVv) are more likely to be recommended than other tests such as skin prick testing for atopy or bronchial challenge testing, because they are cheap and have high positive predictive value, even if the negative predictive value is poor.

Interestingly, a study evaluating the NICE algorithm sequence in children, and a separate study reviewing a similar style of combination testing in adults, both demonstrate a lack of evidence as to the diagnostic reliability of the combination testing algorithms that were utilised [9, 35]. The study in the paediatric cohort used data from the Manchester Asthma and Allergy Study (MAAS), a prospective population-based cohort; the authors demonstrate that the suggested cut-offs which define positive values for spirometry, FeNO and bronchodilator reversibility recommended by NICE were all suboptimal in the cohort of children studied. Moreover, these values are not adjusted for age, height or gender. Cut-offs are the same for all children between 5 and 16 years of age. The authors state that the algorithm should not be used in children. They propose more “realistic” cut-off values for the tests used within the algorithm. [35].

The second study in the adult cohort looked at five diagnostic tests (four of which feature in the NICE guidelines), and the authors demonstrate the difficulties in producing a single sequence to diagnose asthma with both high sensitivity and specificity. They suggest it would be advantageous to first clinically ascertain whether the purpose of the tests is to confirm or exclude asthma [9]. It is important to highlight that both of these studies draw their final conclusions using a “clinical diagnosis” of asthma as the deciding outcome. It is controversial to critique an algorithm using a gold standard that has been criticised as being suboptimal. However, perhaps the take-home message is that more research is required to establish a validated and efficient diagnostic pathway.

## British Guidelines vs International Guidelines

Other national and international guidelines produced or updated over the past decade include Canadian Thoracic Society [30], the Australian Asthma Handbook [8] and the Global Initiative for Asthma (GINA) [6] guidelines. The latter are international guidelines with a focus on managing and diagnosing asthma across all health economies. All of these guidelines recommend that diagnosis include both clinical impression of asthma through a detailed history and examination, and also objective tests. Recommended investigations include spirometry, bronchodilator reversibility, peak flow variability and bronchial challenge testing. None of these guidelines specify the most efficient sequence of tests to best confirm or refute the diagnosis. These guidelines are more in line with NICE recommendations, but in well-defined circumstances will allow a pragmatic diagnosis to be made in the absence of objective tests. The Australian guideline recommends trial of treatment with subsequent diagnosis guided by a suggestion of clinical improvement in children who are unable to perform spirometry. The Canadian guideline also allows for trial of treatment in preschool children. GINA guidelines specify a trial of treatment in anyone whom it is felt there is a more urgent clinical need to commence early treatment. However, there is the expectation that these individuals will return for objective diagnostic testing within 12 weeks. The GINA guidelines also now acknowledge that different subgroups of asthma exist. However, currently they do not recognise a strong enough correlation between the subgroup and the treatment response, and therefore state that tests assessing bronchial hyperresponsiveness or inflammation are not necessary in asthma diagnosis [6]. This contrasts with the emerging approach of sub-grouping asthma into treatable traits [38]. GINA recently updated the Pocket Guide for Asthma Management and Prevention [7] and have included guidance on phenotyping asthma; however, this is not considered until step 5 of the asthma management algorithm, in those whom asthma remains uncontrolled despite high-dose corticosteroids. The potential problem with this approach is that by this stage, the patient has already been subjected to high-dose corticosteroids, which may or may not have been appropriate and also may alter the efficiency of subsequent testing and interpretation of results.

It should be noted that the recommendations for diagnosing asthma in the absence of objective tests in certain patient groups is largely due to a deficiency in tests that can be performed by children. There is a clear need for novel tests that can assess small airway disease in this cohort of the population.

In addition to conflicts regarding the sequence and type of tests recommended across the different guidelines, the threshold used as a positive test also varies (Table 2). The most marked discrepancies appear to be in spirometry, peak expiratory flow variability (PEFv) and exercise challenge testing. For some of these, the differences may appear trivial (e.g. using “ ≥ ” rather than “ > ”), but for others there are significant differences depending on the guideline used (e.g. the lower limit of normal (LLN) for FEV1/forced vital capacity (FVC) for a 20-year-old male is 86%, and for an 80-year-old female is 62%; for neither would a fixed cut-off of 70% be clinically appropriate). Recommendations for PEFv calculations are particularly varied across guidelines.

**Table 2:** Positive test thresholds for objective tests across international guidelines

	BTS [36]	NICE [17]	GINA^a^ [6, 7]
Spirometry	Adults: FEV1/FVC ratio < LLN Children: as above	Adults: FEV1/FVC ratio < 70% (or < LLN if available) Children: as above	Adults: FEV1/FVC < LLN Children: as above
BDR	Adults: FEV1 increase by ≥ 12% and ≥ 200 ml Children: (≤ 16 years): FEV1 increase by ≥ 12%	Adults: FEV1 increase by ≥ 12% and ≥ 200 ml Children: (≤ 16 years): FEV1 increase by ≥ 12%	Adults: FEV1 increase by > 12% and > 200 ml from baseline Children: (6–11 years) FEV1 increase by > 12% of predicted value
FeNO	Adults: ≥ 40 ppb Children: ≥ 35 ppb	Adults: ≥ 40 ppb Children: (≤ 16 years): ≥ 35 ppb	Not included
PEFv	Adults: > 20% variability (using minimum 2-week PEF diary—calculating percentage of the average PEF) Alternatively > 20% variability when symptomatic vs non-symptomatic Children: not recommended	Adults: > 20% variability (using minimum 2-week PEF diary—calculating amplitude as a percentage of mean or highest value) Children: (≤ 16 years) as above	Adults: > 10% variability (using minimum 2-week PEF diary—calculating days highest minus days lowest, divided by mean of days highest and lowest and averaged over the week) Children: (6–11 years) > 13% variability measured as above
BHR tests	Adults: histamine or methacholine PC20 ≤ 8 mg/ml Alternatively mannitol (positive defined as drop in FEV1 > 15%) Children: as above	Adults: histamine or methacholine PC20 ≤ 8 mg/ml Children: (≤ 16 years) not recommended	Adults: histamine or methacholine dose PC20 (guideline states “using standard doses”) Alternatively eucapnic voluntary hyperventilation, hypertonic saline or mannitol PC15 Children: (≤ 16 years) not recommended
Exercise challenge test	Adults: drop in FEV1 > 15% Children: as above	Not included	Adults: drop in FEV1 > 10% and > 200 ml from baseline Children: (≤ 16 years) drop in FEV1 > 12% predicted or PEF > 15%

^a^The GINA 2018 guideline report is used, plus updates have been extracted from the GINA Pocket Guide for Asthma Management and Prevention (updated 2019). The official GINA report for 2019 is not currently available

## The Future of Asthma Diagnosis

Despite some contradictions amongst current asthma diagnostic guidelines, it is clear that the general trend is moving towards diagnosing asthma using objective tests. NICE guidelines are perhaps currently the most aggressive in this approach, driven in part by the consideration of health economics. With the emergence of stratified and biomarker-driven therapeutics, future diagnostics will need to move beyond “asthma”, to enable identification of phenotypes and endotypes. The NICE algorithm is the first to move towards such an approach, by including a non-invasive type II biomarker (high FeNO) that is predictive of corticosteroid responsiveness.

There are specific challenges in achieving an objective diagnosis of asthma in children and adults who cannot perform spirometry or FeNO. However, novel tests of airflow obstruction and airway inflammation (in the small and large airways) are in development and may have an emerging role in asthma diagnosis and phenotyping. Some of these tests are much easier to perform on young children and will potentially enable objective diagnosis of asthma in preschool children (see Table 3) [11].

**Table 3:** Novel tests of airway pathophysiology with future potential in asthma diagnosis

Test	Measures (e.g.)
Impulse oscillometry (IOS) [31]	R5 (total airway resistance at 5 Hz) R20 (central airway resistance at 20 Hz) R5-20 (peripheral airway resistance: the difference between 5 and 20 Hz) X5 (total airway reactance at 5 Hz) AX (reactance area under the curve)
Multiple-breath washout (MBW) [28]	LCI (lung clearance index) Sacin (acinar ventilation heterogeneity) Scond (conductive ventilation heterogeneity)

Novel tests: tests of small airway pathology and inflammation	
Test	Measures (e.g.)
Volatile organic compounds (VOC) [25]	Mass spectrometry Electronic nose
Particles in exhaled air (PExA) [4]	Number of exhaled particles Protein analysis: surfactant protein A, albumin

At present, diagnostic investigations recommended in national asthma guidelines predominantly interpret large airway pathophysiology and fail to take into account the small airways. This is likely due to the ease of access and also minimal invasiveness of large airway tests. Small airways are defined as airways without cartilage and < 2 mm in diameter [39]. Between the trachea and the alveoli there are 23 generations of branching tubes comprising large and small airways [34]. Historically, the small airways have been viewed as a “silent zone” because they account for less than 10% of total airway resistance [12], and until recently, commonly used imaging and physiological tests have not been able to detect abnormalities in these airways. Accurate investigation of the small airways was only possible by invasive procedures such as transbronchial biopsy and post-mortem examination. Non-invasive investigations that can reflect small airways such as forced expiratory flow at 25–75% of pulmonary volume (FEF_25–75_) have been accessible, but the results are highly variable due to its dependence upon the forced vital capacity (FVC) [10]. It has now been accepted that the small airways in patients with asthma are a significant contributor to airflow limitation [12, 14]. Involvement of these airways is not detected by routine spirometry and peak flow monitoring [12]. Using these large airway tests alone may result in missed diagnosis of asthma in patients that have early disease with preserved large airways. It has been demonstrated that pathology can occur in the small airways of patients before changes are detected in spirometry and even before onset of asthma symptoms [34]. Recent advances in non-invasive tests that are able to assess small airway function and composition could potentially enable the detection of asthma at an earlier stage.

Novel tests of small airways include functional tests assessing airway physiology and tests that detect underlying pathology and inflammation. Some promising tests providing information on airway physiology include impulse oscillometry (IOS) [31] and multiple-breath washout (MBW) [28]. In addition, various experimental non-invasive breath analysis tests are emerging and may also have a role in detecting small airway pathology in asthma. Experimental tests include breath composition analysis such as that seen in volatile organic compounds (VOCs) [25] and particles in exhaled air (PExA) [4]. It is likely that we will start to see some of these novel tests incorporated into the asthma diagnostic algorithms over time.

Given the complexity of asthma, it is likely that in the future, a hybrid approach utilising both established and novel tests will be required in the optimal diagnostic pathway. The ultimate goal is to develop a diagnostic pathway that is able to discriminate between both phenotypes and endotypes. However, at this time, underlying endotypes are still being defined, and whilst research continues in this area, it is important to take a more pragmatic approach to diagnosing and treating asthma in the present. There is an urgent clinical need to establish an evidenced-based diagnostic pathway that can identify different subgroups of asthma and identify patients with treatable traits.

The future is likely to see the development of personalised medicine, further enabling the best treatment for each individual patient. The most well-established group of endotypes currently described are type 2 inflammation-associated asthma. The literature reveals that a majority of asthma patients appear to have evidence of type 2 inflammation [18]. This is associated with cytokines (IL4, IL5, IL14) and inflammatory cells (type 2 T helper lymphocytes, mast cells, basophils, eosinophils, IgE-producing plasma cells). Patients with this underlying aetiology respond well to corticosteroids. Attempts to further characterise the predominant molecular pathway have been sought in order to direct a more targeted therapy and reduce the overuse of steroids, which have associated side effects. Type 2 inflammation inhibitors have emerged over the last one to two decades, including drugs that target IgE such as omalizumab, and those that target IL-5 such as mepolizumab, reslizumab and benralizumab [15]. Other potential individualised strategies include bronchial thermoplasty, a technique that uses radiofrequency waves to target smooth muscle and reduce smooth muscle mass in patients that have airway remodelling with smooth muscle hypertrophy and hyperplasia [29].

## Conclusion

The past few decades have seen significant changes in the way we define and diagnose asthma. However, we have yet to establish a unified best practice diagnostic algorithm that not only correctly identifies asthma but also starts to sub-group patients in a way that can signpost them to the most effective treatment pathway. Over the next decade it is likely that we will see the emergence of novel investigations of the small airways enter the asthma diagnostic pathway. In the meantime, it is important to continue to move away from the error-prone “trial of treatment” approach and use existing objective tests to diagnose asthma. What is absolutely clear is that we need to continue to sculpt the current diagnostic and management practice in order to reduce the avoidable morbidity and mortality that are currently associated with asthma.
